# American Dental Association

**Published:** 1871-11

**Authors:** C. Stoddard Smith


### ARTICLE IV.

### *American Dental Association.*

By C. Stoddard Smith, D. D. S.

Second Day—*Morning Session.*

Called to order at 10 o'clock. Minutes read and approved.

The subject of "Dental Physiology" being declared still open—

Dr Taft said he hoped this discussion would not be cut short. He was pleased with the report, yet it seemed scarcely to cover the ground which a report on that subject should cover. Physiology is the science of the functions of living bodies; the report and discussions had had reference to histology—the development of organs. This is an interesting study, but not now under consideration. The manifestations of function are modified by circumstances. All organic tissues do not present the same phases. There are deviations from the normal standard. No two organs are in all respects just alike; there is just as much variety as in the faces of individuals. They have many things in common, but each has individuality. Histology is closely linked with physiology, but the examination of the different organs, their susceptibilities and liabilities, and their influence upon each other, is an important point for our consideration.

Prof. Rufus K. Browne being introduced, said that he would rather speak before this body than one of his own profession. You alone who are advanced and advancing, listen with respective minds. The medical profession, though its backbone has been repeatedly broken, listens with no receptivity. The literature of physiology represents no truth. He finds in that literature one statement alone which represents truth—that in reference to the circulation of the blood, set forth by Harvey. When he began his researches he he did not doubt but that the books contained truth; but eleven years of study, day in and day out, had convinced him that they were a mass of error. Study means, not reading words in a book, but observation of function in the body—the tissue should be studied not the book. The bane of the profession is, that the phrases of books are accounted knowledge. They had better be discarded than leaned upon any longer. The ordinary reader can not pick out the few facts from the thousand errors. Words have no meaning but in the minds of men. The same words which Huxley uses to express the grossest error I can use to express my own convictions of truth—for instance the change of color in the blood. The phrase, as used, represents no truth. What constitutes this change? Physiologists have said that there were two changes; there are no two changes; blood has no color apart from the red globules; they take oxygen into the body, and this produces a change of color. After coursing through the vessels, the blood returns to its normal purple condition; this is called by physiologists another change. The fluids of the blood take no oxygen, the solids only do that; they take up a definite amount, just as is done in the formation of chemical compounds; in doing this the change of color takes place; carbonic acid has no influence in making it blue, though Flint and Dalton will so assert to-day as they have done for years. The moment the oxygen leaves the globule, it leaves it its permanent purple color; carbonic acid does not touch it.

The ruling truth is to see that these globules are what they are, and this truth will one day supplant all error. Will the medical profession aid? Not much. The greatest so-called discoverers are no discoverers; they have literally not the first idea, the new microscopy will differ from the old as truth from error. The relation of oxygen to cells is that of departure and supply.

Dr. Atkinson. We suppose that we are prepared to differentiate between truth and error, in which case we must know more than the authors of books themselves. Facts are the external manifestations of truth.

Prof. McQuillen had noticed with pleasure the enthusiasm manifested by this body when advised to study nature. How many of you will do so? If all that is embodied in works on physiology is as naught, if the labors of men who have been justly regarded as master minds have only resulted in the accumulation of a mass of error, how dark the past! how unpropitious, how hopeless, the future! If the efforts of the past have been barren in their results, how can we expect anything better of the future? Shall we look with contempt on the teachings of the kind mother who starts us on the road to knowledge, because, perchance, one day we may know more than she did? She did her best. Leibig says, "Show me the man who makes no mistakes, and I will show you a man who has done nothing." The fear of making mistakes frequently deters observation and expression of opinion. If he would urge anything on the profession, it would be that its members should make themselves familiar with books and then go to nature. He could not agree with the idea that books should be thrown aside as useless. He does not recognize that any one has taught that oxygen is carried directly to the tissues by the red corpuscles; nutrition is extra vascular.

Prof. Browne. The red corpuscles do not carry oxygen, but yield it to the fluid which carries it. Oxygen nutrifies nothing; substance nutrifies.

Prof. McQuillen repeated that the red corpuscles do not pass out of the capillaries, though in some cases the white corpuscles are said by some observers to do so. Did I understand Prof. Browne to state that no carbonic acid is formed in the tissues?

Prof. Browne. No union of carbon and oxygen takes place in the body. When you state that red corpuscles carry oxygen, you state what the books say, and it is contrary to truth.

Prof. McQuillen. Assertion that fact is fact does not make it so. Men of science demand proof. To deny that there is a union of oxygen and carbon taking place in the body demands more than assertion. Chemists tell us that this does occur. That carbonic acid is present in the venous blood and in the pulmonary exhalations is easily demonstrated. Where does it come from if not from this union? We must consider the conditions. In the dark, certain elements remain in close proximity, without union, for an indefinite period; but let a ray of light enter and they unite with explosive energy. Carbon is found without color in the diamond; in charcoal and graphite it is black as night. Chemically there is little difference in these substances, although so dissimilar in appearance; is it not, then, reasonable to infer that carbonic acid *may* impart color to the blood? Some minds are easily denominated, saying "Yes, yes," to-day, to one man, and "No, no," to another to-morrow, on the same subject. He would impress upon the minds of those present that they should read books but should not be bound down by them; they should respect the efforts of the brilliant minds of the past, and not consider them as ignoramuses, and their works, as containing no truths.

Dr. Harriman. It is our duty to respect brilliant minds, but we must cull over their works. They may have been correct in their day, but science is not stationary. I have assisted Prof. B. in demonstating these facts. He speaks from experience. He has worked hard, and none appreciate it. His book, when it appears, will be a new dispensation. What does a chemist know about living matter? As soon as he examines it he kills it; it is something else. He respects chemistry, but has seen the falsity of many of its teachings as plainly as he sees the President in the chair. The microscope has now been so far improved that the testimony of the fathers is almost set aside.

Prof. McQuillen. The gentleman informs us that he has had peculiar advantages for investigation. I dislike to speak of myself, but would say I have for weeks, from early in the morning till late at night, experimented on this matter of the blood with dogs, cats, rabbits, etc., and do not wish to be understood as speaking from books. These experiments have been republished in the leading medical and microscopical journals of America, England, and Germany. I respect truth whether from a sage or from an ignoramus, and will take exceptions to error emanating from whom it may. Truth will live, and what is the use of foisting in a miserable individuality?

Dr. Atkinson. He who asserts is bound to prove; truth is not negative.

Prof. Judd. That principle is correct, but not always recognized. As to the fact of the circulation of the blood being the only truth in physiology—we are acquainted with the views which have been advanced by Prof. Browne through articles in the journals; but we believe there are other facts beside that one. Does the gentleman mean to assert that the stomach is not a digestive organ, or that the kidneys do not secrete urine? While we recognize his efforts, we know that he is not the only man who has spent his life in the pursuit of science. There is an acquired significance. As to change of color in the blood. That question has been discussed. The gentleman was understood to say that there was but one change of color. There is an admitted change from purple to scarlet. Now, if there is no second change, how does it become purple again? If it is changed, there is a change back again. We are aware that the medical profession has not appreciated the work of investigators; it is the fault of that profession. But there is a growing disposition to admit proof, and when the gentleman's views are proven he will receive credit for all the innovations he has made. The next gentleman endorsed these views with the whole weight of his authority. These errors a longer life will sometimes correct. There are two ways of looking at these things. There are two parties; one is conscious of knowledge that they cannot impart. I am conscious of my own existence, but cannot demonstrate it objectively.

Dr. Harriman. The stomach does not digest the food, it only contains it. The gastric juices assisted by the saliva digest it. As to the white blood corpuscles passing through the walls of the vessels, he has never seen it, but perhaps the previous speaker has. Cells produce cells; he knows that, for he has seen it, as others of you doubtles have.

Dr. Thomas, of Detroit. The questions which have arisen are of great importance; but there are practical questions of more interest. Though he is not a student, yet he has accepted the theories of men whom he sees around him, and is humiliated to hear that no truth is contained in them. The publication of the work referred to would be the proper mode of presenting these views.

The subject was then passed.

The report of the Committee on Dental Pathology was then called for, and Dr. Atkinson, Chairman, read a report of which the following is a very brief synopsis:

It was stated that two questions must be definitely answered before any test basis can be reached: first, what is pathology? and second, what is surgery? We cannot say affirmatively, in honesty, that any such basis has been reached. We have, however, learned much that is affirmative. The habits of the dogs of the chase were spoken of. They endure fatigue without food for days; they refuse food for a time after their return, and eat voraciously subseqently. This is an example of a stage of physiological rest to tissues to fit them for normal work, and this principle has not been sufficiently heeded by physicians and surgeons. The best treatment of surgical cases in fat subjects is to imitate hybernation, and institute forced physiological rest. Supply of nutriment must be fluid, or carried by a fluid to the tissues. Physiology is normal function, pathology is divergent function. It has been hinted that it is impudence to question the authority of books and teachers in our institutions. It is rather the veriest malpractice to follow directions not understood and principles not comprehended. The most advanced are but abecedarians. What, then, shall be said of those who cannot discern the alphabet ? Study *things*, not books ; or if not the thing, its nearest representative, as—1st, its bust; 2d, its portrait, 3d, its description ; and 4th, its name. Surgery is the means made use of to restore lesion of tissues ; it is either conservative or reparative; the former attempts to limit pathological action ; the latter, to restore lost parts.

Cells are endowed with an inner portion of protoplasm, called nucleus, which receives and disposes of the pabulum of the body as the stomach does for the animal. Any disturbing influence to this action produces pain or disease. The perception of this may be central or local ; mind and function are interdependent. Man need not look beyond himself for an acquaintance with the laws which govern his genesis, fruitage, and dispersion in the ocean of force and form, from which were formed the earth and all its inhabitants; so when we have all knowledge pertaining to the least individuality, we shall be able to fulfill our career without arrest. The beginning or ending of individualities will be indifferent as to first or last. The cosmos is a plenum, and must consist of two-ness and not one-ness. Spontaneous origin of tissues is unwillingly acknowledged by the advocates of the "germ" theory. To understand the problem of individual career we must analyze the department of consciousness. We must observe the inception, ripening, fruitage, and dispersion of a single body or process throughout its serial metamorphosis. Thermal changes are motions of molecules. Could we control these (in the body) at will, we should have nutrient activity in our power. Extremes of this condition alike kill the molecules. When the change has not been too great, we restore by a gradual application of heat and cold, and where coagulation has taken place we have recourse to surgical manipulations.

No marked advance has taken place within the year in pathology and surgery. The usefulness of the method of treatment of necrosed bone by the use of sulphuric acid has been more fully established. Two new remedies have been brought into use, viz, thymol, as a substitute for creasote, and the mono-chloro acetic acid, both of which have been imported only a short time. I have tried them, and recommend them to you as invaluable.

Adjourned.

### SECOND DAY—*Afternoon Session.*

Called to order at half-past three o'clock. The Secretary being absent, Dr. C. S. Smith was appointed Secretary *pr tem.*

Prof. McQuillen objected to the resolution adopted yesterday, inviting certain dentists to participate in the discussion, as it was in violation of the constitution and the objects of the association. A reconsideration of the vote by which the resolution was adopted was moved and passed, and the resolution amended so as simply to invite all persons to seats on the floor without participating in the discussions.

"Dental Pathology" being still in order, Dr. Atkinson explained further the new remedy referred to in the report —thymol. He said it was related to the phénol and carbol, series. The objection urged against the smell of creasote does not apply to this. It is rather pleasant.

Dr. Crouse called the speaker to order, on the ground that he was speaking on therapeutics, not pathology. The Chair sustained the point of order.

Dr. Atkinson appealed, and the appeal being sustained

Dr. Atkinson said that some men have to have the grace of God beaten into them with a mallet. Thymol is pleasant to patients, especially hoœopathists, who abhor creasote. It was obtained from Darmstadt, in Germany. I have liked it from the first. It is not fluid at ordinary temperatures, but a glycerole has been prepared which retains its fluidity. I have introduced it and endorsed it fully. It is very fine for ulcerous patches in the mouth. It is a disinfectant.

Prof. McQuillen asked if Dr. Atkinson said that pestilence existed in Persia on account of the absence of scientific knowledge.

Dr. Atkinson. Yes.

Prof. McQuillen said that we are apt to forget our obligations to the East, not only for systems of religion, but also for the development of science, and especially of medicine.

Dr. Atkinson. I said that it was an opportunity to repay the debt we owe these countries, which were formerly lands of religion and science.

Dr. Walker said that he had intended to prepare a report upon the disease produced by rubber, but had not done so. He hoped, however, that the subject will be thoroughly handled. It is well established that the membrane of the mouth is five times as sensitive as any other. The observations of those who do observe carefully establish that this is one of the greatest scourges, and should be discussed thoroughly, and every effort made to get rid of it.

The subject of "Dental Pathology" was then passed.

The Committee on Histology were called and asked for further time. Granted.

The Committee on Voluntary Essays presented a paper by Dr. G. W. Harriman, of Boston, which was read by the author.

The paper opened by asserting that the dentist of the present day must be thoroughly versed in a knowledge of the human frame, and a worker and student in library and laboratory. The variation in the normal number of teeth between the mammalia and other branches of the animal kingdom was noticed. The constituents of the teeth were named—all the dentinal tissues receiving their due supply of lime-salts from the proper sources. There is no such thing as enamel pulp as stated in the books. Enamel is formed by the calcification of the fibres. Prior to calcification, the tissues forming the dentine are a homogeneous mass of fibres and cells. The essayist then took issue with Prof. McQuillen in respect to his views as to the contents of the dentinal tubuli; the latter, as was stated, denying the existence of nerve fibrils in the tubes. In contravention of this doctrine the essayist said, first, that the amount of fibrin in the blood (2 pts. in 1000) was not sufficient, if coagulated, to produce the appearance presented; second, that fibrin will not coagulate when separated from the red corpuscles. After announcing his total want of agreement with authors and investigators, the essayist accepted the commonly accepted theories of the development of the teeth as established by Goodsir, which show that the primitive dental groove, and the germs of the teeth, may be discerned about the sixth week of fœtal existence, asserting that he could discover no osseous deposit in the jaw before the twelfth week, nor any signs of the teeth prior to the seventh month. The essayest scouted the idea that any appendage to a tooth has connected with it anything resembling prisms, pillars, or tubes, hollow columns, tubuli, or hard pipes, as generally in dental works. The tubuli are nothing but cell fibres, branching away from the pulp and terminating at the enamel, and the fact that they are not tubes was said to be established by the impossibility of injecting them with carmine. The paper closed with a reiterated attack upon authorities in general.

Prof. McQuillen said, after the reading of the paper, the ground traveled over obviated the necessity of any report on histology. He may have said positively that dentinal fibrils are coagulated fibrin, but it was not his habit to state things in that manner. He was generally in the habit of offering his views suggestively. He was not as yet convinced that the fibrillæ are nerve fibres. The microscopical appearance of a tooth differs according to whether it is fresh or dry. In the dry tooth there are certainly tubes or tubuli which had been occupied by the soft-solid fibrillæ. The paper said that fibrils existed in the tubes six months after extraction. I ask whether the tooth had been manipulated with chemicals?

Dr. Harriman. Yes.

Prof. McQuillen. Then the dentine was in an altered condition. In making a microscopical examination of a tooth, or any other organized structure, to determine the peculiar characteristics, it should be subjected to as little change of composition as possible. It was stated in the paper that the author had not found teeth germs at any time before the seventh month of uterine existence. Now, it is a historical fact that a king of France, Louis XVI, was born with errupted teeth; cases of this kind are of quite frequent occurrence; this, then, would allow but two months for the processes of tooth formation and eruption. Prof. Judd took some exceptions to the propriety of applying the term exostosis to the enlargement of cementum. He had preparations with him, previously submitted to the Academy of Natural Sciences, in which the difference in bone, dentine, and cementum was apparent—the bone showing Haversian canals, canaliculi, and lacanæ; in hypertrophied cementum the Haversian canals are not found. A man accustomed to the microscope can readily distinguish between hypertrophied comentum and bone. As to nerve fibrils in the tubuli, he must see them to be convinced—it was his practice to follow assertions with demonstration. He wanted to see not only the nerve fibres, but the nerve cells, that had been described and figured in an article published in the *Dental Cosmos.*

Dr. Atkinson. There is evidently a boggle about the conception of truth. Exceptions may be taken to the paper in a number of its parts. The gentleman though a faithful worker, shows a want of range of observation. Bone, enamel, and cement have different characteristics. How shall we recognize them? This depends on the locality of the bone. A single stratum might contain no Haversian canals; but if you have enough to say that it is bone, you have a Haversian system. This system is a portion of cartilage that has been converted into bone. If the uncalcified tracts are large enough, they have blood vessels running through them. Cement is the medium of communication of pronouncements of force and form. The give-and-take movement is constantly going on on all sides of the little lake. Cement has canaliculi, never Haversian canals. The corpuscles of cement are never equally distributed around the canaliculi; those next the pabulum have more branches than on the side nearest the enamel. What is dentine? A compromise between cementum and enamel. Uncalcified tracts are not solid tracts.

Dr. Crouse moved that an extra session be held to-morrow evening, for the purpose of fixing the place of next meeting and election of officers. Carried.

Adjourned.

*To be continued.*

---

